# A Network Biology Framework for Exploring Molecular Mechanisms in Cystoid Macular Edema

**DOI:** 10.22336/rjo.2026.09

**Published:** 2026

**Authors:** Mehrdad Motamed Shariati

**Affiliations:** 1 Eye Research Center, Mashhad University of Medical Sciences, Mashhad, Iran

**Keywords:** cystoid macular edema, protein-protein interaction network, ANGPT2, VEGFA, inflammation, angiogenesis, systems biology, enrichment analysis, VEGFA = Vascular Endothelial Growth Factor A, ANGPT2 = Angiopoietin-2, KDR = Kinase Insert Domain Receptor (VEGFR-2), FLT1 = Fms Related Tyrosine Kinase 1 (VEGFR-1), PDGFB = Platelet-Derived Growth Factor Subunit B, IL6 = Interleukin 6, TNF = Tumor Necrosis Factor, CXCL8 = C-X-C Motif Chemokine Ligand 8 (IL-8), CXCL10 = C-X-C Motif Chemokine Ligand 10, CCL2 = C-C Motif Chemokine Ligand 2 (MCP-1), ICAM1 = Intercellular Adhesion Molecule 1, EDN1 = Endothelin 1, HIF1A = Hypoxia Inducible Factor 1 Subunit Alpha, NOS2 = Nitric Oxide Synthase 2 (Inducible), MMP2 = Matrix Metallopeptidase 2, MMP9 = Matrix Metallopeptidase 9, TGFB1 = Transforming Growth Factor Beta 1, PTGS2 = Prostaglandin-Endoperoxide Synthase 2 (COX-2), IL10 = Interleukin 10, IL4 = Interleukin 4, IL13 = Interleukin 13, EPO = Erythropoietin, TGFBR1 = Transforming Growth Factor Beta Receptor 1, TGFBR2 = Transforming Growth Factor Beta Receptor 2, ICAM2 = Intercellular Adhesion Molecule 2, VCAM1 = Vascular Cell Adhesion Molecule 1, STAT3 = Signal Transducer and Activator of Transcription 3, MAPK1 = Mitogen-Activated Protein Kinase 1 (ERK2), MAPK3 = Mitogen-Activated Protein Kinase 3 (ERK1), MAPK8 = Mitogen-Activated Protein Kinase 8 (JNK1), MAP2K1 = Mitogen-Activated Protein Kinase Kinase 1, RELA = RELA Proto-Oncogene, NF-κB Subunit, NFKB1 = Nuclear Factor Kappa B Subunit 1, NFKBIA = NFKB Inhibitor Alpha, FGF2 = Fibroblast Growth Factor 2, FGF1 = Fibroblast Growth Factor 1, FGFR1 = Fibroblast Growth Factor Receptor 1, FGFR2 = Fibroblast Growth Factor Receptor 2, CDH5 = Cadherin 5 (VE-Cadherin), CLDN5 = Claudin 5, OCLN = Occludin, JAM2 = Junctional Adhesion Molecule 2, S1PR1 = Sphingosine-1-Phosphate Receptor 1, AQP4 = Aquaporin 4, SOD2 = Superoxide Dismutase 2, Mitochondrial, NOS3 = Nitric Oxide Synthase 3 (Endothelial), CYBA = Cytochrome B-245 Alpha Chain, NOX4 = NADPH Oxidase 4, PECAM1 = Platelet and Endothelial Cell Adhesion Molecule 1, SELE = Selectin E, SELP = Selectin P, ITGB2 = Integrin Subunit Beta 2, ITGAM = Integrin Subunit Alpha M, CCR2 = C-C Motif Chemokine Receptor 2, CCL5 = C-C Motif Chemokine Ligand 5 (RANTES), CXCL9 = C-X-C Motif Chemokine Ligand 9, IL1B = Interleukin 1 Beta, IL1A = Interleukin 1 Alpha, IL17A = Interleukin 17A, IL18 = Interleukin 18, IFNG = Interferon Gamma, SOCS3 = Suppressor of Cytokine Signaling 3, JAK1 = Janus Kinase 1, JAK2 = Janus Kinase 2, TYK2 = Tyrosine Kinase 2, IL6R = Interleukin 6 Receptor, IL10RA = Interleukin 10 Receptor Subunit Alpha, IL4R = Interleukin 4 Receptor, IL13RA1 = Interleukin 13 Receptor Subunit Alpha 1, PI3KCA = Phosphatidylinositol-4,5-Bisphosphate 3-Kinase Catalytic Subunit Alpha, AKT1 = AKT Serine/Threonine Kinase 1, AKT2 = AKT Serine/Threonine Kinase 2, FOXO1 = Forkhead Box O1, BCL2 = BCL2 Apoptosis Regulator, BAX = BCL2 Associated X, Apoptosis Regulator, CASP3 = Caspase 3, CASP9 = Caspase 9, MMP1 = Matrix Metallopeptidase 1, MMP3 = Matrix Metallopeptidase 3, MMP7 = Matrix Metallopeptidase 7, TIMP1 = TIMP Metallopeptidase Inhibitor 1, TIMP2 = TIMP Metallopeptidase Inhibitor 2, PLAT = Plasminogen Activator, Tissue Type, PLAU = Plasminogen Activator, Urokinase, SERPINE1 = Serpin Family E Member 1 (PAI-1), HMOX1 = Heme Oxygenase 1, PRKCB = Protein Kinase C Beta, PRKCA = Protein Kinase C Alpha, ERK1 = Extracellular Signal-Regulated Kinase 1 (MAPK3), ERK2 = Extracellular Signal-Regulated Kinase 2 (MAPK1), RAC1 = Rac Family Small GTPase 1

## Abstract

**Objectives:**

To explore the molecular mechanisms underlying cystoid macular edema (CME) through protein-protein interaction (PPI) network analysis, identifying key regulatory proteins, functional modules, and enriched biological pathways relevant to its pathogenesis.

**Methods:**

A curated list of 18 CME-associated human proteins was compiled through a literature review, including inflammatory cytokines, angiogenic factors, and regulators of vascular permeability. High-confidence PPI data were obtained from the STRING database (confidence score ≥ 0.700) and visualized as an undirected graph using NetworkX in Python. Centrality metrics (degree, betweenness, closeness, eigenvector), Louvain modularity analysis, and functional enrichment (GO: BP, KEGG, Reactome) were performed using GSEAPY and Enrichr to identify topologically and biologically important nodes and clusters.

**Results:**

The final network consisted of 91 nodes and 217 edges, with a graph density of 0.0505 and an average clustering coefficient of 0.0554. ANGPT2, FLT1, KDR, TNF, and VEGFA appeared as central hub proteins across multiple centrality measures. Louvain clustering identified eight distinct functional communities, including inflammatory mediators, angiogenic signaling components, and tight junction regulators. Enrichment analyses showed significant involvement of cytokine-mediated signaling, endothelial cell migration, the AGE-RAGE pathway, TNF signaling, and interleukin-4/10/13 signaling, highlighting the dual roles of angiogenesis and inflammation in CME.

**Discussion:**

The findings of this study highlight the intricate network of molecular mechanisms underlying cystoid macular edema (CME), with particular emphasis on angiogenesis, inflammation, and vascular permeability. The identification of key regulatory proteins such as ANGPT2, FLT1, and TNF underscores the complexity of CME pathogenesis, which involves both vascular and inflammatory pathways.

**Conclusions:**

CME is driven by a complex, modular molecular network involving inflammation, vascular remodeling, and cytokine signaling. ANGPT2, FLT1, TNF, and IL6 are key regulatory proteins and potential therapeutic targets. This systems biology approach offers a comprehensive framework for discovering new insights and guiding personalized treatment strategies in CME.

## Introduction

Cystoid macular edema (CME) is a retinal condition characterized by fluid accumulation in cystoid spaces within the macula, leading to visual impairment. CME often occurs as a complication of various eye diseases, including diabetic retinopathy, uveitis, retinal vein occlusion, and postoperative inflammation. The main process behind CME involves breaking down the blood-retinal barrier (BRB), increasing blood vessel permeability, and causing inflammation and changes in retinal structure. Although treatments such as corticosteroids, anti-VEGF agents, and nonsteroidal anti-inflammatory drugs are used, CME frequently recurs and can be resistant to therapy, making it a significant clinical challenge [1,2].

While individual molecules such as vascular endothelial growth factor A (VEGFA), interleukins, and matrix metalloproteinases have been linked to CME, the disease’s pathophysiology goes beyond these isolated pathways. It involves complex molecular interactions among many proteins that promote inflammation, angiogenesis, immune cell recruitment, and BRB dysfunction. Understanding these detailed molecular relationships is essential for discovering new therapeutic targets and improving existing treatment strategies [3,4].

Protein-protein interaction (PPI) networks offer a systems-biology approach to understanding the organizational principles underlying disease-related molecular mechanisms. By depicting biological systems as networks of interacting proteins, PPI analysis provides insights into key regulatory hubs, important signal transduction mediators, and functional modules [5].

This network-centric perspective helps identify proteins of high topological significance that could serve as potential biomarkers or therapeutic targets in complex disorders such as CME.

In this study, we constructed and analyzed a PPI network associated with CME using high-confidence interaction data retrieved from the STRING database. We selected a curated panel of CME-associated proteins based on a literature review, including VEGFA, IL6, TNF, ICAM1, MMPs, chemokines, and tight junction regulators [6-8].

We evaluated the network’s topological characteristics using multiple centrality metrics, performed a modularity analysis with the Louvain algorithm, and conducted pathway enrichment analyses to infer functional relevance. This integrated approach sheds light on the molecular landscape of CME, highlighting critical proteins and biological processes that may inform future mechanistic studies and therapeutic developments.

## Materials and methods

### Literature-Based Identification of CME-Associated Proteins

To establish a biologically grounded foundation for network analysis, we began with a comprehensive literature review to identify proteins implicated in the pathophysiology of cystoid macular edema (CME). Searches were conducted using PubMed and Scopus databases with combinations of the following keywords: “cystoid macular edema”, “inflammation”, “angiogenesis”, “vascular permeability”, “cytokine”, “growth factor”, and “hypoxia”. Proteins were considered for inclusion if there was experimental or clinical evidence linking them to CME or related ocular diseases such as diabetic retinopathy, retinal vein occlusion, or uveitis. Only human protein-coding genes were considered, and gene symbols were verified via the UniProt and GeneCards databases. The final curated list of 18 seed proteins spanned multiple mechanistic domains, including angiogenesis (VEGFA, ANGPT2, KDR, FLT1, PDGFB), inflammation and cytokine signaling (IL6, TNF, CXCL8, CXCL10, CCL2), vascular permeability (ICAM1, EDN1), oxidative stress and hypoxia (HIF1A, NOS2), matrix remodeling (MMP2, MMP9), and additional regulators such as TGFB1 and PTGS2 [9-13].

### Retrieval of Protein-Protein Interaction Data

Protein-protein interaction (PPI) data for the seed proteins were obtained from the STRING database (version 12, https://string-db.org/), using its RESTful API and the Python requests library. The analysis was limited to *Homo sapiens* (taxonomy ID 9606), and only interactions with a combined STRING confidence score of 0.700 or higher were included to ensure high reliability. The returned dataset included interaction partners, preferred gene names, and combined interaction scores, integrating data from experimental results, curated databases, co-expression patterns, and computational predictions.

### Network Construction and Visualization

The retrieved interaction data were used to build a weighted, undirected graph using the NetworkX library in Python. In this graph, each node represented a protein, while each edge indicated a high-confidence interaction between two proteins, weighted by the STRING combined score. Isolated proteins (with no high-confidence interactions) were excluded from the final network. The resulting graph was visualized using spring layout algorithms to organize spatial relationships and served as the basis for topological and functional analyses.

### Topological Characterization of the Network

To assess the structure and connectivity of the CME-associated protein network, we calculated several centrality and global network metrics. Degree centrality was used to identify hub proteins with the highest number of direct connections. Betweenness centrality quantified the extent to which proteins acted as bridges along the shortest paths between other nodes. Closeness centrality was used to evaluate how quickly information could spread from a node to others. Eigenvector centrality measures a protein’s influence based on the importance of its neighbors. All centrality metrics were normalized to the range 0-1, with higher values indicating greater relative importance or connectivity within the network. Local clustering coefficients are calculated to assess the tendency of proteins to form tightly interconnected groups. Global metrics, including graph density, average clustering coefficient, network diameter, and average shortest path length (computed over the largest connected component), provided a broader view of network architecture and navigability.

### Community Detection and Functional Clustering

To uncover potential biological subsystems within the network, we employed the Louvain community detection algorithm via the community (python-louvain) package. This unsupervised method optimizes network modularity by grouping nodes into non-overlapping communities with dense internal connections. Each node was assigned to a community, and the number, size, and connectivity of the resulting modules were analyzed to detect functionally coherent clusters. These communities potentially correspond to biological pathways or co-regulated protein complexes relevant to CME.

### Functional Enrichment Analysis

The biological significance of the proteins in the extended network was evaluated using gene set enrichment analysis (GSEA) via the Enrichr platform, accessed through the GSEAPY Python package (version 1.0.5). The full set of proteins (including first-degree interactors from STRING) was submitted to three annotation databases: Gene Ontology Biological Process 2021 (GO: BP), KEGG 2021 Human, and Reactome Pathways 2022. Enrichment significance was determined using Fisher’s exact test, and multiple comparisons were adjusted using the Benjamini-Hochberg method. Gene sets with adjusted p-values less than 0.05 were considered statistically significant. For the top enriched terms, overlap statistics and adjusted p-values were reported, and –log10-transformed adjusted p-values were visualized as bar plots to highlight the most relevant biological processes and pathways.

### Computational Environment and Reproducibility

All analyses were conducted in a Python 3.10 environment using Jupyter Notebook for interactive development and documentation. The primary computational libraries included NetworkX for graph modeling and analysis, community for modularity detection, GSEAPY for enrichment analysis, and Matplotlib/Seaborn for data visualization. STRING API requests were handled using the requests package, and data wrangling was performed with Pandas and NumPy. The entire computational pipeline was version-controlled and documented to ensure reproducibility. The full source code and instructions for replicating the analysis are available at: https://github.com/MehrdadMotamed-lab/CME_PPIN/blob/main/CME_PPIN2.ipynb.

## Results

### Network Construction and General Topology

**Fig. 1:**
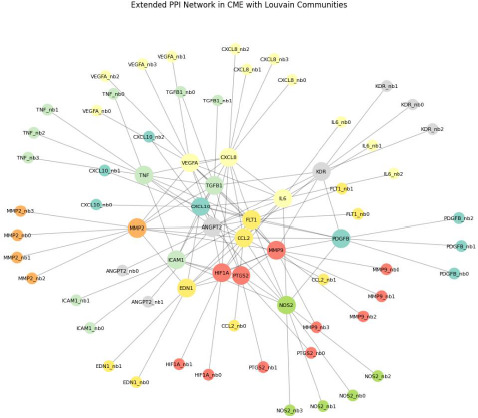
The protein-protein interaction network of cystoid macular edema

The final protein-protein interaction (PPI) network, constructed using 18 seed proteins and their high-confidence first-shell interactors from the STRING database, consisted of **91 nodes** and **217 edges (**Fig. 1**)**. Each node represents a protein, and edges indicate interactions with a STRING combined confidence score ≥ 0.700. The network exhibited a **graph density of 0.0505**, reflecting a sparsely connected but biologically relevant interaction structure. The **average clustering coefficient** was **0.0554**, suggesting a modest tendency for proteins to form local clusters. The **diameter** of the largest connected component was **4**, and the **average shortest-path length was 2.97, indicating efficient communication among** proteins within the network (**Table 1**).

**Table 1:** Network analysis results

Network Statistics	
	Number of nodes: 91 Number of edges: 217 Graph density: 0.050 Average clustering coefficient: 0.055
Top 5 by Degree Centrality	
	ANGPT2: 0.2097 FLT1: 0.1935 TNF: 0.1774 KDR: 0.1774 VEGFA: 0.1452
Top 5 by Betweenness Centrality	
	ANGPT2: 0.1899 FLT1: 0.1629 KDR: 0.1456 CXCL8: 0.1399 PTGS2: 0.1329
**Top 5 by Closeness Centrality**	
	ANGPT2: 0.4769 FLT1: 0.4662 KDR: 0.4627 VEGFA: 0.4429 TNF: 0.4397
**Top 5 by Eigenvector Centrality**	
	ANGPT2: 0.3315 FLT1: 0.3011 KDR: 0.2964 TNF: 0.2920 HIF1A: 0.2605

### Centrality Metrics Identify Key Regulatory Proteins

Quantitative topological analysis was performed to identify proteins of high regulatory significance. According to **degree centrality**, which measures the number of direct connections a node has, the top five proteins were **ANGPT2 (0.2097)**, **FLT1 (0.1935)**, **TNF (0.1774)**, **KDR (0.1774)**, and **VEGFA (0.1452)**. These proteins serve as network hubs, potentially controlling information flow across multiple biological modules.

**Betweenness centrality**, which captures the extent to which a protein lies on the shortest paths between other proteins, identified **ANGPT2 (0.1899)**, **FLT1 (0.1629)**, **KDR (0.1456)**, **CXCL8 (0.1399)**, and **PTGS2 (0.1329)** as major bridging proteins. These nodes are likely involved in coordinating distinct signaling pathways relevant to CME pathogenesis.

**Closeness centrality**, indicating the speed at which a protein can influence the entire network, was highest for **ANGPT2 (0.4769)**, **FLT1 (0.4662)**, **KDR (0.4627)**, **VEGFA (0.4429)**, and **TNF (0.4397)**. These findings suggest that angiogenic regulators and inflammatory cytokines are optimally positioned to propagate signals throughout the CME network rapidly.

In terms of **eigenvector centrality**, which measures a node’s influence based on the connectivity of its neighbors, the most central proteins were **ANGPT2 (0.3315)**, **FLT1 (0.3011)**, **KDR (0.2964)**, **TNF (0.2920)**, and **HIF1A (0.2605)**. These results further underscore the functional importance of these molecules, particularly in the context of angiogenesis, inflammation, and hypoxia-related signaling.

### Modular Structure Reveals Distinct Functional Communities

Using the Louvain community detection algorithm, the network was partitioned into **eight non-overlapping communities**. These included clusters ranging from **five to fourteen nodes**, indicating modular organization within the CME interactome. The largest community (14 nodes) predominantly included cytokine receptors and inflammatory mediators, while other communities captured proteins involved in angiogenic signaling, endothelial migration, matrix remodeling, and tight junction regulation. This modular arrangement reflects the multifactorial nature of CME pathophysiology, encompassing interlinked biological systems.

### Functional Enrichment Highlights Inflammatory and Angiogenic Mechanisms

**Fig. 2:**
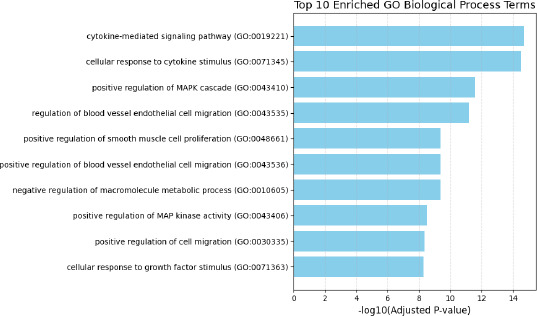
Functional enrichment analysis. GO: Gene Ontogeny

Gene ontology and pathway enrichment analysis were conducted on the full network to reveal underlying biological processes. Analysis of the **GO: Biological Process (2021)** dataset identified several highly significant terms. The top five enriched processes included **cytokine-mediated signaling pathway** (*adjusted p* = 1.94×10^−^^15^; 14/621 genes overlapped), **cellular response to cytokine stimulus** (*adjusted p* = 3.21×10^−^^15^; 13/482), **positive regulation of MAPK cascade** (*adjusted p* = 2.78×10^−^^12^), **regulation of blood vessel endothelial cell migration** (*adjusted p* = 6.60×10^−^^12^), and **positive regulation of smooth muscle cell proliferation** (*adjusted p* = 4.07×10^−10^). These terms suggest coordinated regulation of immune activation, intracellular signaling, and vascular remodeling (**Fig. 2**).

In the **KEGG 2021 Human** pathway analysis, the most enriched pathways were the **AGE-RAGE signaling pathway in diabetic complications** (*adjusted p* = 8.28×10^−^^15^; 9/100), **fluid shear stress and atherosclerosis** (*adjusted p* = 8.78×10^−14^), **rheumatoid arthritis** (*adjusted p* = 2.95×10^−^^13^), **TNF signaling pathway** (*adjusted p* = 1.03×10^−^^12^), and **pathways in cancer** (*adjusted p* = 2.94×10^−^^12^). These pathways collectively highlight the intersection of systemic inflammation, vascular dysfunction, and chronic disease in CME.

**Reactome Pathway enrichment** analysis further reinforced the dominance of cytokine and interleukin signaling. The most significant terms were **interleukin-4 and interleukin-13 signaling** (*adjusted p* = 2.76×10^−19^; 11/107), **signaling by interleukins** (*adjusted p* = 1.92×10^−14^), **interleukin-10 signaling** (*adjusted p* = 2.76×10^−^^13^), **cytokine signaling in the immune system** (*adjusted p* = 1.80×10^−^^12^), and **signal transduction** (*adjusted p* = 1.01×10^−8^). These results confirm the central role of cytokine networks in orchestrating the inflammatory environment of CME.

## Discussion

Cystoid macular edema (CME) is a multifactorial and vision-threatening condition resulting from the pathological accumulation of fluid within the macular region of the retina. It often arises as a complication of various retinal and systemic diseases, including diabetic retinopathy, retinal vein occlusion, uveitis, and postoperative inflammation [13]. Despite the availability of anti-VEGF therapies, corticosteroids, and NSAIDs, CME frequently presents therapeutic resistance and recurrence, underscoring the need for a deeper understanding of its molecular underpinnings [14]. In this study, we applied a systems-biology approach centered on protein-protein interaction (PPI) network analysis to investigate the molecular mechanisms and functional architecture of CME. By integrating curated literature-based protein selection, network modeling using STRING and NetworkX, topological analysis, community detection, and pathway enrichment, we delineated the complex molecular landscape that underlies CME pathophysiology.

Our network comprised 91 nodes and 217 high-confidence edges, representing both seed proteins and their interactors. The graph density (0.0505) and average clustering coefficient (0.0554) indicated a moderately sparse yet biologically meaningful structure, suggesting that CME-related proteins do not operate in isolation but instead form specialized interaction modules. This architecture reflects the inherently complex and modular nature of chronic inflammatory and vascular retinal diseases.

Centrality analysis provided a key insight into the topological prominence of several proteins, most notably **ANGPT2, FLT1, KDR, TNF, VEGFA, and IL6**. **ANGPT2**, or angiopoietin-2, emerged as the most influential protein across all major centrality metrics, including degree, betweenness, closeness, and eigenvector centrality. ANGPT2 is a context-dependent antagonist or agonist of the TIE2 receptor and plays a pivotal role in vascular destabilization and endothelial permeability, both hallmarks of CME [15]. Its high degree and betweenness indicate its broad connectivity and regulatory potential, acting as a molecular bridge between angiogenesis and inflammatory modules [16]. These findings align with reports showing elevated vitreous ANGPT2 levels in patients with diabetic macular edema and other neovascular retinal diseases [17,18].

Closely trailing ANGPT2 in centrality were **FLT1 (VEGFR-1)** and **KDR (VEGFR-2)**—two key receptors for VEGFA. The inclusion of both receptors among the top central nodes reinforces the centrality of the VEGF signaling axis in CME, particularly in its vascular leakage phenotype. Notably, **VEGFA** itself showed a high degree and closeness centrality, further supporting the hypothesis that this growth factor operates at the center of the CME molecular network [19,20]. These observations support the continued therapeutic relevance of anti-VEGF agents in CME treatment and suggest that VEGF-independent nodes, such as ANGPT2, may serve as alternative or adjunct therapeutic targets, particularly in cases refractory to anti-VEGF therapy.

Interestingly, **TNF** and **IL6**—two well-established inflammatory cytokines—also ranked highly in network centrality. TNF showed high betweenness and eigenvector centrality, suggesting its role as a critical hub linking inflammatory and vascular dysfunction modules [21,22]. Similarly, **IL6**, though slightly lower in the overall centrality rankings, remains biologically significant due to its documented involvement in uveitis-associated CME and response to IL6 inhibitors such as tocilizumab [23]. Together, these findings reaffirm the dual contribution of **angiogenic** and **inflammatory signaling axes** in CME, consistent with its variable clinical presentation and response to therapy across different etiologies.

Community detection using the Louvain algorithm revealed eight distinct functional modules, each potentially representing discrete biological programs contributing to CME. The largest community contained key cytokines, interleukins, and signaling intermediates, suggesting an inflammatory core within the network. Other communities were composed of angiogenic mediators, tight junction proteins, and hypoxia response factors, suggesting that CME arises from the convergence of multiple biological subsystems rather than a single linear pathway. This modular structure echoes findings from retinal transcriptomic studies, which have identified simultaneous activation of immune, oxidative, and angiogenic signatures in eyes with edema [24-26].

Functional enrichment analysis further corroborated these insights. Among the **top Gene Ontology biological processes**, cytokine-mediated signaling and cellular responses to cytokines were the most significantly enriched, followed by MAPK cascade regulation and endothelial cell migration. These results indicate that inflammation, cytokine signaling, and endothelial remodeling form a triad of key biological processes driving CME. Enrichment of the **AGE-RAGE signaling pathway** in KEGG, especially in the context of diabetic complications, underscores the relevance of advanced glycation and oxidative stress pathways in diabetes-associated CME. This is particularly relevant given the clinical burden of diabetic macular edema and its often-suboptimal response to standard therapies.

The enrichment of **fluid shear stress and atherosclerosis**, **TNF signaling**, and **rheumatoid arthritis pathways** further supports a systemic inflammatory influence on the retinal microenvironment. These findings highlight potential shared mechanisms between systemic autoimmune diseases and retinal inflammation, suggesting that CME may act as a sentinel marker of systemic immune dysregulation in certain patients.

Reactome analysis provided additional granularity, highlighting **interleukin-4**, **interleukin-10**, and **interleukin-13 signaling** pathways as among the most significantly enriched. These interleukins are central to Th2-mediated immune responses and vascular homeostasis [27]. For instance, **IL-4 and IL-13** are known to disrupt endothelial barrier integrity and modulate macrophage polarization, both of which may contribute to CME formation [28]. The prominence of **IL-10 signaling**, an anti-inflammatory cytokine, suggests an endogenous counter-regulatory response to ongoing inflammation in the retinal microenvironment [29]. The Reactome category “cytokine signaling in the immune system” was also strongly enriched, again reinforcing the idea that CME exists within a tightly regulated immune signaling landscape.

Collectively, these findings present a compelling case for considering CME not merely as a localized ocular phenomenon but as the product of integrated and systemic molecular dysregulation. The strong network connectivity and pathway enrichment of proteins involved in inflammation, angiogenesis, vascular remodeling, and oxidative stress support a multifactorial pathophysiology that varies with the underlying disease etiology. This has therapeutic implications. While anti-VEGF therapy remains a cornerstone for many CME cases, our network analysis identifies several alternative targets—most notably ANGPT2, IL6, and TNF—which may warrant therapeutic consideration, especially in refractory or recurrent CME.

Moreover, this systems-level approach provides a framework for identifying biomarker candidates. Proteins with high centrality metrics may serve as accessible indicators of disease activity or treatment response, particularly if their expression correlates with clinical severity. For instance, aqueous or serum levels of ANGPT2 or TNF could be evaluated longitudinally in patients undergoing anti-VEGF or steroid therapy to assess predictive value.

However, this study is not without limitations. The network was constructed using high-confidence interactions from the STRING database, which, while comprehensive, is inherently static and does not account for tissue-specific expression, temporal dynamics, or post-translational regulation. Moreover, the PPI model reflects potential physical or functional interactions, not necessarily active pathways in CME-affected tissue. Thus, future work should incorporate **multi-omics data**—including single-cell RNA-sequencing and phosphoproteomics—from retinal tissues to refine network relevance. Experimental validation of hub protein expression and interaction dynamics in CME models will also be essential to translate these findings into therapeutic applications.

## Conclusion

In summary, our integrative PPI network analysis reveals a complex but coherent molecular architecture underlying cystoid macular edema. The prominence of ANGPT2, FLT1, VEGFA, TNF, and IL6 underscores the importance of both angiogenic and inflammatory signaling pathways. Modular and enrichment analyses point to distinct yet interrelated processes that drive disease, including cytokine-mediated inflammation, endothelial permeability, and tissue remodeling. These insights may inform the development of more personalized and effective therapeutic strategies for CME, especially for patients unresponsive to conventional treatments. Our findings highlight the value of systems biology in ophthalmic disease research and open new avenues for translational investigation into CME.

## Data Availability

The data supporting this study’s findings are available from the corresponding author upon reasonable request.
